# Chloroquine-Induced Fixed Drug Eruption: A Case Report

**DOI:** 10.7759/cureus.69063

**Published:** 2024-09-10

**Authors:** Yashasvi Anand, Sanket S Bakshi, Swaroopa Chakole

**Affiliations:** 1 Department of Medicine, Jawaharlal Nehru Medical College, Datta Meghe Institute of Higher Education and Research, Wardha, IND; 2 Department of Dermatology, Dr. Vithalrao Vikhe Patil Foundation's Medical College and Hospital, Ahmednagar, IND; 3 Department of Community Medicine, Jawaharlal Nehru Medical College, Datta Meghe Institute of Higher Education and Research, Wardha, IND

**Keywords:** drug reaction, hypersensitivity, topical steroids, violaceous rash

## Abstract

Recurrent erythematous rashes in the same anatomical site are a feature of a disorder known as fixed drug eruption (FDE). We describe a rare instance of a fixed eruption in a 70-year-old woman who had recurrent skin lesions at the same locations due to the use of chloroquine. This case demonstrates the timely and precise identification of an FDE, which resulted in the offending medication being stopped and the symptoms clearing up when topical steroids were used. This case study underscores the significance of attentive healthcare practices and the vital impact that thorough, individualized treatment plays in improving the patient's condition.

## Introduction

Skin manifestations known as “fixed drug eruptions” (FDEs) are linked to the use of specific medications. There are many medications linked to FDEs. For example, certain antiepileptics like phenytoin have been the culprits in certain cases. Nonsteroidal anti-inflammatory drugs are the most common culprit, along with other antibiotics such as ampicillin and metronidazole. Since they are less prevalent among medication responses, they have several variations and are generally unknown to doctors. They are frequently misdiagnosed or confused for bug bites, urticaria, or other disorders [1]. Due to the numerous variations of the illness, these cutaneous drug eruptions may present as various clinical presentations, but they often take the form of oval, erythematous patches. They appear on the skin of the face, feet, tongue, hand, chest, upper extremities, lower extremities, and genital organs [2]. Since they frequently reoccur in the same spot as prior responses, FDE should be considered when there has been a history of lesions in the same area in the past [3]. Although the lesions are benign, their appearance, pruritus, and discomfort are the chief complaints of the patient. When it comes to treating an FDE, a thorough history and physical examination can help identify the culprit drug and stop the administration promptly.

## Case presentation

**Figure 1:**
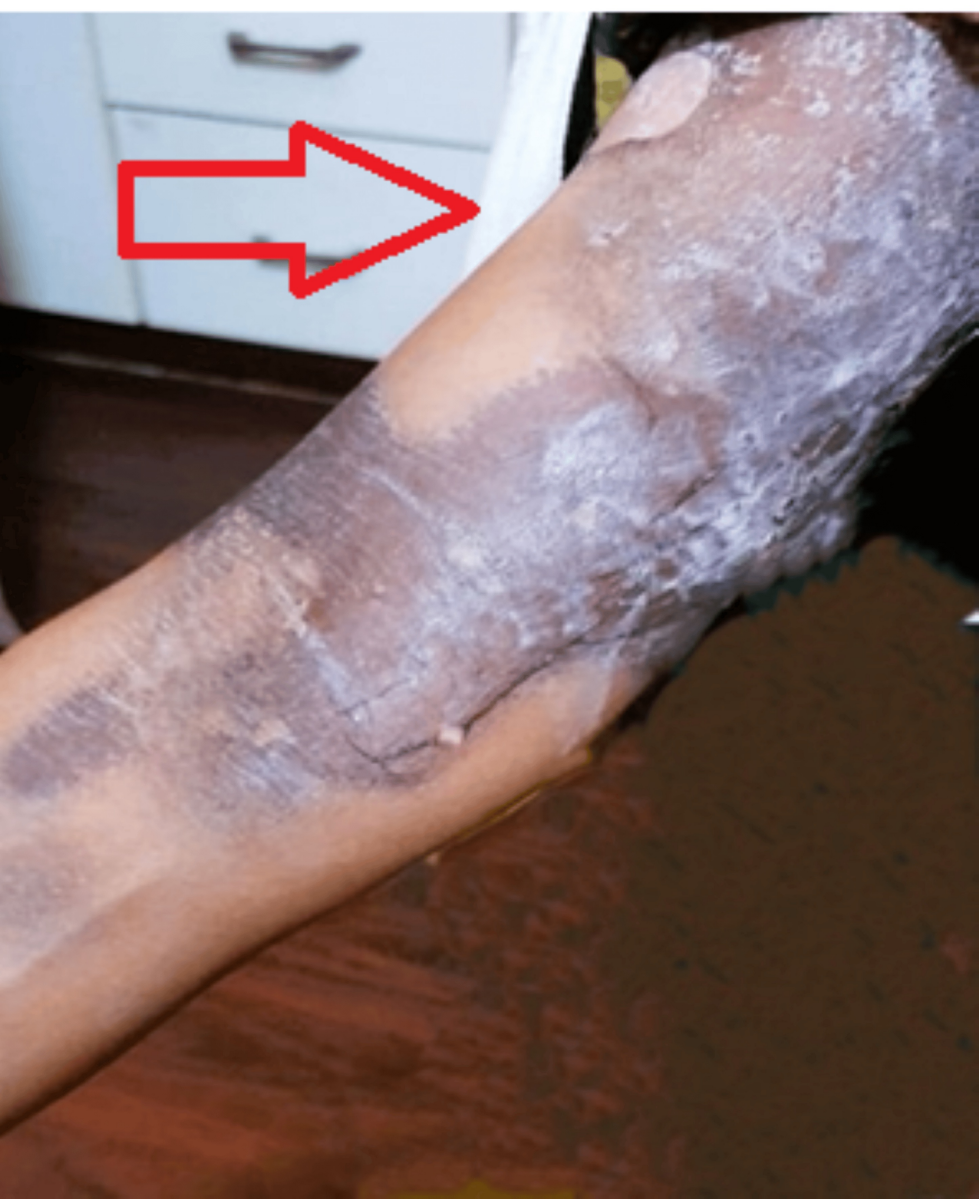
Extensive violaceous patch-like lesion (red arrow)

A 70-year-old female patient arrived at our emergency department with pruritic, violaceous skin lesions that had been on the left extensor aspect of her leg for four days and on the proper inner aspect of her right arm and forearm for two days. Two days before the onset of the lesions, a history of 1,000 mg stat dosage of chloroquine was consumed, followed by 500 mg about eight hours later. After a detailed examination, it was determined that the patient had malaria, for which chloroquine had been prescribed. The oral and vaginal mucosae have never experienced erosion before. The patient was a known case who had previously succumbed to a drug response after receiving nonsteroidal anti-inflammatory medicines, for which the patient was provided with a list of prospective substances that may induce similar drug reactions in the future. On cutaneous examination, the patient had a large violaceous to the erythematous plaque of discoloration on the inner aspect of the right arm with a large bulla on the medial aspect, and an area of skin detachment/erosion is also appreciated on the outer side. The content of the blister seems clear fluid, as shown in Figure 1.

**Figure 2:**
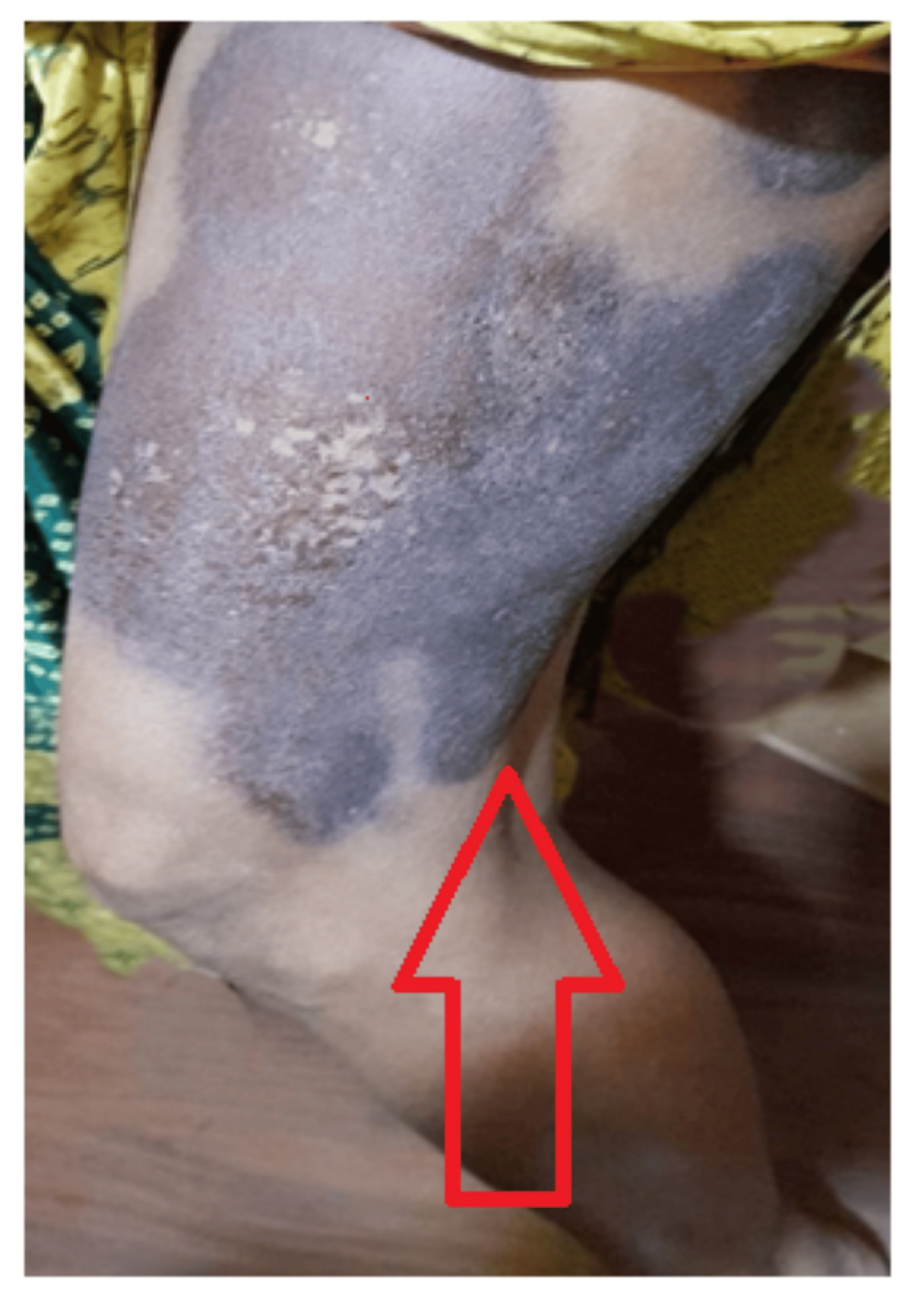
Nondischarging, flat, extensive patch (red arrow)

A 15 x 6 cm flat, non-discharging plaque with a central violaceous area and a peripheral erythematous halo was seen over the right extensor aspect of the thigh, as depicted in Figure 2.

**Figure 3:**
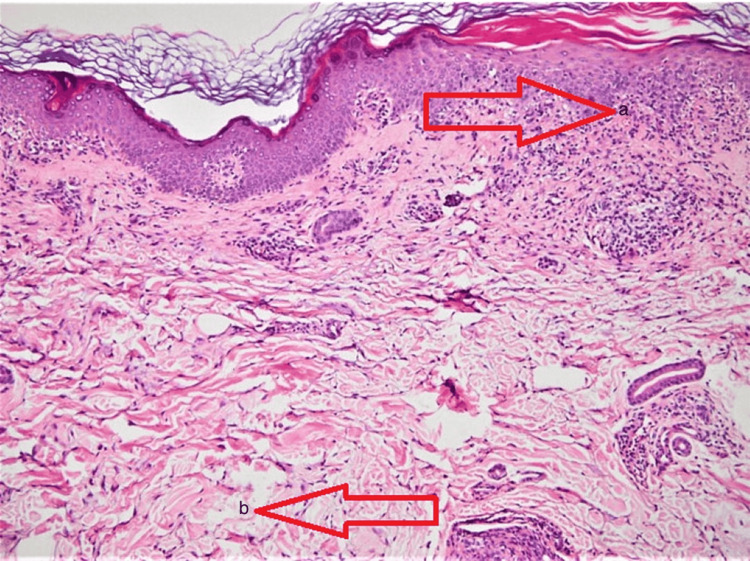
Histopathological picture depicting (a) epidermal dyskeratosis and (b) basal layer degeneration (red arrows)

Figure 3 depicts a 4 mm skin punch biopsy performed in this case to confirm the clinical diagnosis. Histopathological findings showed hydropic degeneration in the basal layer, dyskeratotic cells in the upper epidermis, and inflammatory infiltrate.

Other anatomical sites were regular, with no obvious pathology noted. The Naranjo causality score of 8 indicated that chloroquine was the “possible” cause of the response. We diagnosed FDE related to chloroquine based on the previous conclusive reports, the appearance of adverse effects after administration of a suspected drug, conventional clinical presentation, and high Naranjo score. The culprit medicine was promptly stopped, and the patient was administered topical clobetasol propionate 0.1% w/w and oral H2 blockers as a therapy strategy. The lesions regressed with persistent hyperpigmentation during the follow-up assessment.

## Discussion

An unpropitious reaction to administering certain drugs, leading to the appearance of erythematous papules with specific characteristic findings on histopathology, is known as a fixed drug reaction [4]. After reexposure to the causative chemical, these lesions repeat at the same site. However, other locations may be implicated in some instances [5,6]. FDE accounts for around 10% of cutaneal adverse medication responses and dominates places such as the face, extremities, and genitals [3,7]. The adverse reaction reappears within a few hours when the drug is readministered and dissipates within 10-20 days, leaving a hyperpigmented region behind [8]. Type IV hypersensitivity reaction with inflammation and tissue injury mediated by CD4+Th1 and cytotoxicity of CD8+cytotoxic T lymphocytes at particular locations is hypothesized as the pathogenesis. Antiepileptics such as barbiturates, nonsteroidal anti-inflammatory drugs like paracetamol and aspirin, and antibiotics such as cotrimoxazole, tetracyclines, atenolol, and sulphonamides are the most commonly implicated medications [9]. To our knowledge, there have been just a few previous instances of FDE caused by chloroquine. Although there is no known ethnic or gender preference for FDEs, a single study suggested that the gene human leukocyte antigen B22 may have a genetic relationship with the development of the response. The prognosis is favorable, and most patients recover completely after discontinuing the offending substance [5]. After the reaction has resolved, postinflammatory hyperpigmentation is seen, which is resolved and usually goes away within a few months. The primary goal of FDE management should be to identify and discontinue the problematic medicine. Symptomatic management includes topical corticosteroids and antihistamines, which can give symptomatic relief; however, doctors should remain careful when using levocetirizine and cetirizine because both antihistamines have been linked to FDEs.

## Conclusions

FDEs are dermatological symptoms of a medication-induced response. A history and physical examination typically make the diagnosis; however, knowledge of the various clinical presentations would assist in a timely diagnosis. After administering a new medicine, an alarming situation like a similar previous episode in the same region should alert the doctor to the diagnosis. Postinflammatory hyperpigmentation is common in patients, and it usually resolves with time. FDEs are less prevalent among medication responses, have various varieties, and are typically unknown to primary care providers; hence, there is a need to enhance awareness of this illness. Prevention is essential because an FDE cannot be reversed, and the pigmentation might last forever. This can be accomplished by increased understanding of the most prevalent causal medicines, the risk of recurrence with the same or comparable drugs, and using alternatives when possible.
